# Blood-Contacting Biomaterials: *In Vitro* Evaluation of the Hemocompatibility

**DOI:** 10.3389/fbioe.2018.00099

**Published:** 2018-07-16

**Authors:** Marbod Weber, Heidrun Steinle, Sonia Golombek, Ludmilla Hann, Christian Schlensak, Hans P. Wendel, Meltem Avci-Adali

**Affiliations:** Department of Thoracic and Cardiovascular Surgery, University Hospital Tübingen, Tübingen, Germany

**Keywords:** hemocompatibility, blood contact, biomaterials, coagulation, complement system

## Abstract

Hemocompatibility of blood-contacting biomaterials is one of the most important criteria for their successful *in vivo* applicability. Thus, extensive *in vitro* analyses according to ISO 10993-4 are required prior to clinical applications. In this review, we summarize essential aspects regarding the evaluation of the hemocompatibility of biomaterials and the required *in vitro* analyses for determining the blood compatibility. Static, agitated, or shear flow models are used to perform hemocompatibility studies. Before and after the incubation of the test material with fresh human blood, hemolysis, cell counts, and the activation of platelets, leukocytes, coagulation and complement system are analyzed. Furthermore, the surface of biomaterials are evaluated concerning attachment of blood cells, adsorption of proteins, and generation of thrombus and fibrin networks.

## Introduction

Hemocompatibility is one of the major criteria, which limit the clinical applicability of blood-contacting biomaterials. These materials come in close contact with blood, which is a complex “organ,” comprising of 55% plasma, 44% erythrocytes, and 1% leukocytes and platelets. Thus, adverse interactions between newly developed materials and blood should be extensively analyzed to prevent activation and destruction of blood components. The initially adsorbed protein layer on the biomaterial surface mainly triggers the adverse reactions, such as the activation of coagulation via intrinsic pathway, the activation of leukocytes, which results in inflammation, and the adhesion and activation of platelets (Liu et al., 2014). As a result, the number of blood cells can decrease and a thrombus can be formed.

Thus, the applied blood-contacting biomaterials should not adversely interact with any blood components and activate or destruct blood components. Erythrocytes are the most abundant blood cells with 4–6 × 10^6^ cells/μl and they are important for the transport of oxygen (O_2_) from the lung to all tissues and cells and carbon dioxide (CO_2_) from tissues back to the lung. Since erythrocytes are the most rigid cells in the blood, they are sensitive to rupture and hemolysis due to shear stress and changes in osmotic pressure. Blood platelets are the smallest (1–3 μm) and the second abundant cell type in the blood with 1.5–3.5 × 10^5^ cells/μl, which can rapidly recognize foreign surfaces and initiate blood coagulation. Furthermore, human blood contains 4.3–10 × 10^3^ leukocytes/μl, such as granulocytes, lymphocytes, monocytes, dendritic and natural killer cells. Monocytes account for 1–6% of all leukocytes and neutrophil granulocytes are the most abundant leukocytes in the blood, comprising 50–70% of all leukocytes. These immune cells are belonging to the innate immune system and they can be rapidly activated upon recognition of a foreign invader such as a pathogen or a foreign material. Furthermore, blood plasma contains high amounts of plasma proteins, such as albumin, coagulation factors, and immunoglobulins.

**Figure 1:**
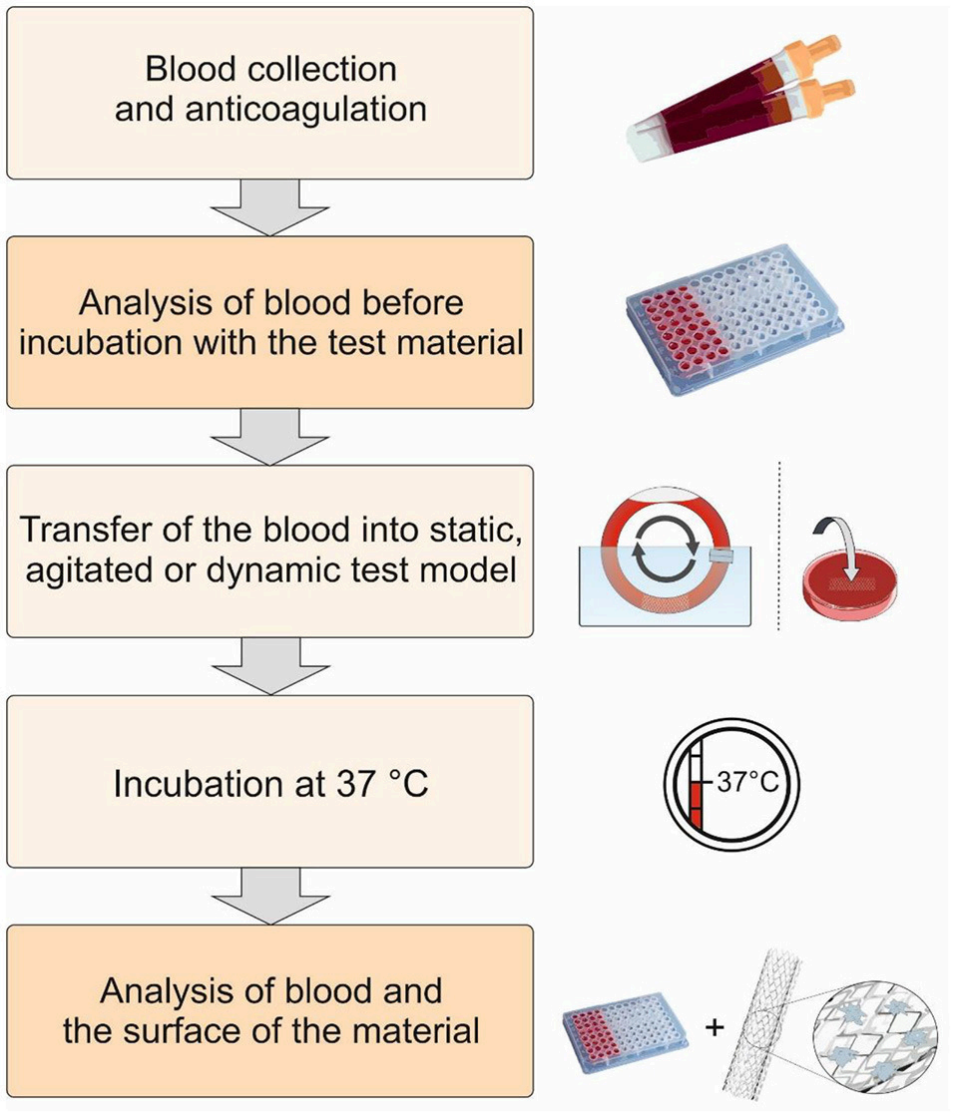
Schematic representation of the procedure for the evaluation of the hemocompatibility of biomaterials. First, fresh human blood is collected and anticoagulated with low dose heparin. Thereafter, the test material is incubated at 37°C using static, agitated, or dynamic test models with the blood. The activation markers in the blood are analyzed before and after the incubation with the test material. Furthermore, the surface of the biomaterial is analyzed to determine the interaction of blood cells and proteins with the biomaterial surface.

Catheters, guidewires, dialyzer, oxygenators (artificial lungs), heart-supporting systems, cardiac pacemaker, vascular grafts, stents, heart valves, micro-, and nanoparticles are widely used medical devices and materials coming in direct contact with blood. Prior to clinical application, the hemocompatibility of blood-contacting medical materials have to be analyzed and therefore, a guidance is developed by the International Organization for Standardization (ISO 10993-4) (International Organization for Standardization, 2000). According this guideline, five different categories, thrombosis, coagulation, platelets, hematology, and immunology (complement system and leukocytes), are indicated for hemocompatibility evaluation. The devices are divided into three categories concerning blood contact: (1) Externally communicating devices with indirect blood contact, e.g., cannulas and blood collection sets; (2) Externally communicating devices with direct blood contact, e.g., catheters and hemodialysis equipment; (3) Implant devices, e.g., heart valves, stents, and vascular grafts. So far, several studies were performed according ISO-10993-4 to evaluate various blood contacting devices and materials, such as stents (Sinn et al., 2011; Stang et al., 2014), catheters with a noble metal alloy coating (Vafa Homann et al., 2016), poly(2-dimethylamino-ethylmethacrylate) (PDMAEMA) (Cerda-Cristerna et al., 2011), and DNA hydrogels (Stoll et al., 2017). To perform hemocompatibility analysis, static, agitated, or shear flow *in vitro* models are used for the incubation of blood with the biomaterial. Before and after the incubation of biomaterials with fresh human blood, the activation markers regarding hemocompatibility are analyzed (Figure 1).

## Incubation of biomaterials with human blood

Using fresh human blood and adequate *in vitro* models, the hemocompatibility of blood-contacting biomaterials can be studied accurately. Compared to *in vivo* animal models, *in vitro* models allow the analysis under well controllable conditions such as blood flow, anticoagulation and eliminate disturbing factors related to flow obstruction, surgery, and tissue effects (van Oeveren, 2013). Furthermore, the blood contact is more intense and products generated due to reaction of the blood components to the biomaterial are not cleared. Moreover, using *in vitro* models, different devices can be analyzed under the same conditions, which enable the direct comparison of outcomes. Thereby, positive controls, which show a poor hemocompatibility, such as glass (Ferrer et al., 2013), devices or biomaterials, which are already on the market with a comparable surface area, and negative controls without test material should be simultaneously tested to be able to evaluate the hemocompatibility.

The quality of collected blood is extremely important to enable standardized hemocompatibility analysis. The *in vitro* analysis should be performed with fresh blood from healthy subjects (Blok et al., 2016). Blok et al. demonstrated that the stationary storage of blood over 4 h at room temperature affects the platelet function and activity of leukocytes. Thus, experiments should be started within 4 h after the blood collection. However, the faster the experiments are started, the better it is. Peripheral blood should be collected from healthy non-smoker, non-pregnant subjects free of medication (particularly drugs affecting the hemostasis, such as aspirin, oral contraceptives, and nonsteroidal anti-inflammatory drugs). Moreover, atraumatic blood collection by minimizing venostasis during blood withdrawal and the use of 21-gauge needles is required to minimize activation of platelets and the coagulation cascade during collection (Braune et al., 2013).

Furthermore, prior to starting hemocompatibility analyses, unreacted monomers, intermediate- or by-products, solvents, and unwanted chemical residues should be removed by appropriate washing and cleaning procedures from generated biomaterials to eliminate an unwanted influence on blood components. Depending on biomaterial, device, and production technique, different washing and cleaning procedures with different solutions are required, for example ethanol can be removed by evaporation or washing with PBS (Punet et al., 2015), NaCl, or water and unreacted methacrylic anhydride and by-products can be eliminated by dialysis (Xiao et al., 2011; Camci-Unal et al., 2013). Additionally, endotoxin content should be determined to exclude material-unrelated activation of platelets and blood cells due to presence of endotoxins (Watanabe et al., 2003; Kälsch et al., 2007; Schrottmaier et al., 2016). Furthermore, a process called depyrogenation can be applied to remove endotoxins from biomaterials (Li and Boraschi, 2016). For example by using detergents (e.g., Triton X-114) and a two-phase extraction method, endotoxins can be eliminated (Zhang et al., 2015). After the addition of the detergent to the sample, endotoxins are incorporated into micelles via non-polar interactions of the surfactant end groups and alkyl chains of lipid A, which is the most conserved part of endotoxins (Magalhães et al., 2007). The increase of temperature leads to the formation of a water phase (micelle-poor) and a micelle-rich phase. Thereby, endotoxins remained in the micelle-rich phase (bottom-phase) can be removed.

### Static blood incubation models

In static blood incubation models, test materials are incubated with blood or platelet-rich plasma (PRP) without flow conditions (Mohan et al., 2013). Therefore, container, such as well plates or tubes, can be used to incubate the material with a certain blood or PRP volume. It is a very simple and rapid method to determine the hemocompatibility of biomaterials, especially the thrombogenicity. However, this method provides only rudimentary results regarding hemocompatibility. Thereby, major limitations can be the cell sedimentation and the big blood-air interface, which can lead to a protein aggregation and result in platelet activation (Haycox and Ratner, 1993).

### Agitated blood incubation models

In agitated blood incubation models, flat incubation chambers with top and bottom surfaces made of the test material are completely filled with blood and incubated on a shaker or overhead rotator without directed flow (Streller et al., 2003). Furthermore, the container filled with the blood can be rotated to prevent the sedimentation of the test material, such as nanoparticles (Maitz et al., 2017). Using these models, the blood-air contact is almost excluded and cell sedimentation is reduced.

### Shear flow models

Flat-plate flow chambers (Van Kruchten et al., 2012), parallel-plate and cone-platelet viscometer (Lackner et al., 2012), and tubular systems such as the “Chandler loop” (McClung et al., 2007; Krajewski et al., 2013; Stang et al., 2014) and the roller pump closed-loop (Podias et al., 1995; Wang et al., 2001) systems are some of *in vitro* shear flow models. In these models, vascular blood flow is mimicked to simulate the dynamic interaction between the biomaterial and whole blood (Sanak and Wegrzyn, 2010). In flat-plate flow chambers, blood flows over a flat piece of biomaterial and in parallel-plate viscometers, blood is filled between two plates made of the biomaterial to be tested and one of the plates is rotated relative to the other (Sukavaneshvar, 2017). In Chandler loop, a circular conduit made of the biomaterial or coated with the biomaterial, is filled with fresh human blood with air bubble to allow the blood mixture and rotated in a water bath with 37°C to stimulate blood circulation. In modified versions of the Chandler loop, small materials, such as stents are inserted in tubings and then filled with blood (Müller et al., 2012). Since the Chandler loop is partially filled with air, the device circulates through an air-liquid interface. Thus, this method can lead to the denaturation of proteins at the air-liquid interface (Thorsen et al., 1993; Ritz-Timme et al., 1997) and the detachment of adhered blood cells. Therefore, roller pump closed-loop test systems were also used instead of Chandler loop. Hereby, the blood flow is realized by using a pump. However, due to use of a pump even with lowest pumping rates a slight destruction of erythrocytes (hemolysis) can occur. Van Oeveren and colleagues analyzed additionally to the Chandler loop and roller pump model, the Hemobile model (Van Oeveren et al., 2012) regarding intrinsic damage of blood components and activation of platelets. Hemobile model has a one-way ball valve to ensure unidirectional flow and the tubing contains no air, and there is no mechanical device compressing the tubing. Thus, using this model, less blood trauma was induced compared to Chandler-Loop and roller pump model.

However, the main limitation of these *in vitro* models is the lack of an endothelium in the circulating system. The endothelium produces cytokines, anti-thrombotic components and expresses adhesion molecules for thrombocytes, monocytes, and neutrophils and plays an important role in interaction between the circulating blood and injured vessel wall. Therefore, in a recent study, Nordling et al. used a novel blood endothelial cell chamber model to study the interactions between human whole blood and endothelium (Nordling et al., 2014). There, the blood contacting surface of incubation chambers were seeded with human umbilical vein endothelial cells (HUVECs). Furthermore, a relatively new field for the examination of platelet and coagulation activation is the use of microfluidics (Kent et al., 2010; Onasoga-Jarvis et al., 2013, 2014; Kovach et al., 2014; Zhu et al., 2015; Nagy et al., 2017). Using microfluidic flow devices platelet and coagulation activation can be determined at the same time and under defined, physiological or pathological (stenotic) wall shear rates. Due to the small size of microfluidic devices, only small amounts of blood are required. Furthermore, the combination with fluorescence microscopy allows the real-time optical imaging of platelet adhesion and formation of fibrin fibers (Westein et al., 2012; Zhu et al., 2015).

## Analysis of hemocompatibility

**Figure 2:**
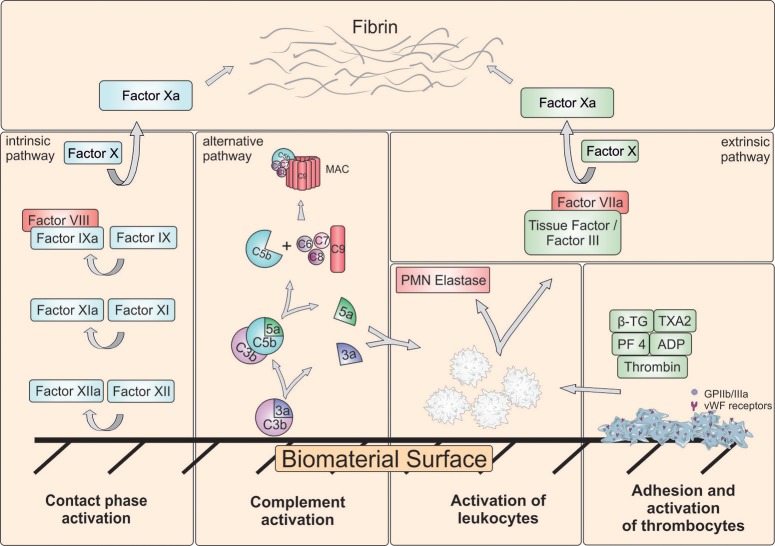
Schematic representation of major reactions in blood induced by biomaterial surface. Besides complement system, intrinsic and extrinsic coagulation pathway can be activated. Coagulation activation results in generation of a fibrin network. Furthermore, platelets can adhere and aggregate on the surface. The adhesion and activation of leukocytes can lead to the release of polymorphonuclear (PMN) elastase and tissue factor (TF) and result in activation of extrinsic pathway. ADP, Adenosine diphosphate; β-TG, β-Thromboglobulin; GPIIb/IIIa, Glycoprotein IIb/IIIa; MAC, Membrane attack complex; PF4, Platelet factor 4; TXA2, Thromboxane A2; vWF, von Willebrand factor.

Using the described *in vitro* models, various information regarding hemocompatibility (Figure 2) can be obtained: (1) Changes of platelets, erythrocytes and leukocytes, (2) Generation of activation products in plasma, (3) Deposition of proteins and cells on the material surface. Thus, blood and surface of biomaterials are analyzed before and after the incubation. In Table 1, test categories for the hemocompatibility analysis of biomaterials are summarized and in the following, the required analyses for the evaluation of hemocompatibility are described.

**Table 1:** Summary of test categories for the hemocompatibility analysis of biomaterials.

**Test category**	**Parameter**	**Test principle**
Complement System	C3a, C5a, Bb, C4d, C5b-9	ELISA
Coagulation	Factor XIIa, TAT, F1 + 2, free active thrombin, FPA, aPTT	ELISA, Optical density, Viscoelasticity
Fibrinolysis	D-Dimers	Immunoturbidimetry, LPIA, ELISA
Platelets	β-TG, PF4, number of platelets, P-selectin, activated GPIIb/IIIa	ELISA, Cell Counter, FACS
Hemolysis	Number of erythrocytes, Hemoglobin	Cell Counter Colorimetric Assay
Leukocyte Activation	PMN elastase, ROS detection, CD11b expression	ELISA, fluorimetric or spectrophotometric methods, FACS
Surface Analysis	Platelet adhesion, aggregation, leukocyte adhesion Plasma protein adsorption	SEM, Fluorescence microscopy ELISA, Western Blot

*aPTT, activated partial thromboplastin time; β-TG, β-Thromboglobulin; C3a, Complement factor 3a; C5a, Complement factor 5a; C4d, Complement factor 4d; Bb, complement factor Bb; ELISA, Enzyme-linked Immunosorbent Assay; FACS, Fluorescence-activated cell sorting; FPA, fibrinopeptide A; F1+2, Prothrombin fragment 1+2; LPIA, Latex photometric immunoassay; PF4, Platelet factor 4; TAT, thrombin-antithrombin III complex; PMN elastase, Polymorphonuclear elastase; ROS, Reactive oxygen species; SEM, Scanning electron microscopy*.

## Determination of blood cell numbers and hemolysis

The number of erythrocytes, leukocytes, and platelets is measured before and after the incubation of blood with biomaterial using a hematology analyzer, which uses electrical impedance. A decrease of the platelet count over time indicates a thrombogenic material. Furthermore, the rupture of erythrocytes, called hemolysis, is accompanied by the release of hemoglobin. Thus, an increased concentration of free hemoglobin in the plasma is a direct indicator of erythrocytes destruction. The damage of erythrocytes can lead to the reduced oxygen transport to tissues and organs *in vivo* and increased levels of free hemoglobin can induce toxicity or alter the kidney function (Qian et al., 2010). Additionally, microvesicles derived from erythrocytes can promote the thrombus formation in a tissue factor (TF)-dependent manner (Biró et al., 2003). Thus, hemolysis can be analyzed after direct or indirect blood contact. In direct analysis, blood is incubated with the biomaterial and in indirect testing blood is incubated with biomaterial extract (Kuhbier et al., 2017). Hemolysis can be detected by using a photometric colorimetric test (cyanmethemoglobin method) (Stadie, 1920). Thereby, the free amount of hemoglobin in plasma is examined after the addition of cyanmethemoglobin (CMH) reagent, e.g., Drabkin's reagent, which rapidly converts hemoglobin to the cyanoderivate (Neun and Dobrovolskaia, 2011). The absorption of CMH is measured at 540 nm using a photometer. Depending on hemolysis, materials can be classified in three different categories: Materials resulting in over 5% hemolysis are classified hemolytic, between 5 and 2% as slightly hemolytic, and below 2% as nonhemolytic (Totea et al., 2014).

## Coagulation activation

The interaction of plasma proteins with artificial surfaces triggers intrinsic coagulation pathway by contact activation. The contact-phase system consists of three serine proteinases, factor XII, factor XI, and plasma prekallikrein (PK), and the nonenzymatic cofactor high molecular weight kininogen (HMWK) and it is also called as plasma kallikrein-kinin system (Wu, 2015). The contact of blood with artificial, negatively charged surfaces, such as kaolin, glass, collagen, silica, or dextran sulfate, leads to the conversion of Factor XII to the active enzyme Factor XIIa. Factor XIIa converts PK into active kallikrein and HMWK into bradykinin. Besides coagulation, kallikrein and bradykinin promote inflammation (Long et al., 2016). Kallikrein can directly activate neutrophils (Wachtfogel et al., 1995) and bradykinin can stimulate the release of nitric oxide (Bae et al., 2003), TNFα, and IL-1 (Tiffany and Burch, 1989). Factor XIIa activates Factor XI to XIa and in the following step, Factor IX is converted by Factor XIa to IXa, which then activates factor X. The conversion of factor X into factor Xa is the first common step in the coagulation cascade between the intrinsic and extrinsic pathways (Millar et al., 2016). In addition, the activation of complement system can lead to the generation of TF by monocytes, which can result in activation of extrinsic pathway (Kappelmayer et al., 1993). Factor Xa converts prothrombin to thrombin, which hydrolyses fibrinogen into fibrin and leads to the subsequent clot formation.

The activation of coagulation system is screened by detection of FXIIa (Basu et al., 2017), prothrombin fragment 1 + 2 (F1 + 2) (Maitz et al., 2017; Sperling et al., 2017), which is released during thrombin formation, free active thrombin (Müller et al., 2011), fibrinopeptide A (FPA) (Peckham et al., 1997), partial thromboplastin time (PTT), or thrombin-antithrombin III complex (TAT). Furthermore, the degradation product of fibrin, D-dimer, can be detected by ELISA to determine the activation of fibrinolysis (Sperling et al., 2017). Antithrombin III inhibits thrombin by forming a TAT complex. Thus, this complex reflects a functional state of the coagulation system and can be quantified using ELISA. PTT assay measures the clotting time from the activation of Factor XII to the formation of a stable fibrin clot. To detect PTT, citrated platelet-poor plasma is incubated at 37°C with the test material for 15, 30, or 60 min. The addition of PTT reagent (cephalin) followed by the addition of calcium chloride solution inactivates the anticoagulant and initiates clot formation and this time is recorded to obtain the activated PTT (aPTT). A shortened clotting time indicates an activation of the intrinsic and common pathways of coagulation by the test material. Untreated plasma is used as negative control and latex or black rubber as positive control.

## Activation of complement system

Complement system consists of more than 30 proteins circulating in the blood and present as membrane-associated proteins (Dunkelberger and Song, 2010). In response to the recognition of foreign surface structures, complement factors are sequentially activated in an enzyme cascade via three different pathways: classical, alternative, and mannose binding lectin (MBL) pathway. All of these pathways lead to the generation of a C3 convertase, which cleaves C3 into a large fragment C3b, which acts as an opsonin and a small fragment C3a, which is an anapylatoxin that promotes inflammation. Afterwards, C5 convertase is generated, which cleaves C5 in C5a, which is an anapylatoxin, and C5b that binds to the foreign surface and initiates the generation of terminal lysis complex (C5b-9, TCC), which is also called membrane attack complex (MAC). As a result, microorganisms are eliminated by lysis, opsonization and triggering a series of inflammatory reactions.

The contact of the artificial surface with blood leads to an immediate adsorption of serum proteins, e.g., fibrinogen, albumin, and immunoglobulin G (IgG), to the surface of the material (Wetterö et al., 2000) and it results in a kinetic competition between the proteins on the material surface, which is called the Vroman effect (Vroman et al., 1980). During the first minutes, abundant proteins, such as fibrinogen, adsorb and they are progressively displaced by less abundant proteins with a higher affinity for the surface, such as HMWK, Factor XII, and plasminogen (Ballet et al., 2010). Especially, the complement protein C3 and IgG can readily bind to hydrophobic surfaces and lead to the activation of complement system. Thereby, the following conformational changes on the blood-contacting surface are considered as initial trigger for the complement activation via the alternative or classical pathway (Gorbet and Sefton, 2004; Andersson et al., 2005; Nilsson et al., 2007). Different biomaterial surfaces show different complement activating properties, for example, hydrophobic surfaces can lead to an increased complement activation compared to hydrophilic surfaces (Nilsson et al., 2007).

The generated complement activation products lead to the increased expression of P-selectin, which is an important mediator of neutrophil recruitment and platelet accumulation (Sukavaneshvar, 2017). Compared to C3a and C4a, the produced C5a is the most powerful anaphylatoxin. It can increase the permeability of blood vessels, attract and activate neutrophil granulocytes and monocytes to stimulate phagocytosis. C5a stimulates endothelial cells to increasingly express cytokines, chemokines and cell adhesion molecules, such as E-selectin (Newton and Dixit, 2012). Furthermore, it can bind to mast cells and increase inflammation. Moreover, C5a is able to trigger the release of TF from neutrophils and monocytes, which can initiate coagulation cascade (Ikeda et al., 1997; Ritis et al., 2006; Kourtzelis et al., 2010). Thus, there is a close cross-talk between the complement system and the coagulation pathway mediated by the generated C5a (Oikonomopoulou et al., 2010).

Therefore, the analysis of complement activation is a highly relevant criterion in the legislation for testing biomaterials intended for blood contact. According to ISO 10993-4 (International Organization for Standardization, 2000), the complement activation can be analyzed by detection of C3a, C5a, Bb, iC3b, C4d, C3-, or C5-convertase, and the C5b-9 complex in whole blood, as well as the 50% complement hemolytic activity (CH50) (Costabile, 2010). Most frequently, the concentration of anaphylatoxins C3a and C5a as well as the C5b-9 complex is determined using ELISA (Kopp et al., 2002; Sperling et al., 2007; Engberg et al., 2011). Furthermore, in a recent study, Endgberg and colleagues proposed that the ratio between the binding of C4 and its inhibitor C4BP should be considered as a predictor for the evaluation of the hemocompatibility (Engberg et al., 2015).

## Platelet activation

Platelets are present in large quantities in the blood and under physiological conditions, they circulate in a quiescent state for 7–10 days. The adhesion and activation of platelets is prevented by an healthy endothelial monolayer, which acts as a barrier between blood and tissues and has antithrombogenic properties by the release of e.g., nitric oxide (NO) and prostaglandin I_2_ (PGI_2_) (Brass et al., 2013; Golebiewska and Poole, 2015; Frohlich, 2016). The damage of endothelium leads to the exposure of the underlying subendothelial collagen to the blood. In addition, damaged endothelial cells secrete von Willebrand factor (vWF), which can bind to the collagen in the exposed subendothelial layer and mediate the adhesion of circulating platelets (Yau et al., 2015) to form a seal at the damaged area.

The exposure of biomaterial to the blood can result in an undesired activation of platelets and consequently lead to thrombotic complications. Thus, the analysis of platelet activation is an important part of hemocompatibility tests. The contact of blood with foreign surfaces immediately leads to the adsorption of plasma proteins, especially fibrinogen, immunoglobulins, fibronectin, vitronectin, Factor XI and XII, vWF, HMWK, and PK to the biomaterial surface (Long et al., 2016). Particularly, fibrinogen, vWF, fibronectin, and vitronectin induce the adhesion of platelets via interaction with the most frequent integrin receptor α_IIb_β_3_ glycoprotein IIb/IIIa (GPIIb/IIIa) on the surface of platelets and lead to the activation of platelets. Subsequently, platelets release bioactive molecules from their alpha and dense granules. Each platelet contains ~50–80 alpha granules and ~3–6 dense granules (Fitch-Tewfik and Flaumenhaft, 2013). The dense granules contain proaggregatory factors such as adenosine diphosphate (ADP), adenosine triphosphate (ATP), histamine, serotonin [5-hydroxytryptamine (5-HT)], polyphosphates, and bivalent cations Mg^2+^ and Ca^2+^ (Meyers et al., 1982). ADP can activate neighboring platelets via binding to two different purinergic receptors on platelets, known as P2Y_1_ and P2Y_12_ (Wijeyeratne and Heptinstall, 2011). These platelets can activate further platelets passing by and lead to the adhesion and aggregation of neighbored platelets. Finally, the thrombi is stabilized by fibrin. Alpha granules contain hemostatic proteins, such as vWF, fibrinogen, Factor V, Factor IX, and plasminogen, chemokines (e.g., platelet factor 4 (PF4), SDF-1α), and growth factors (e.g., VEGF, PDGF). Furthermore, large amounts of β-thromboglobulin (β-TG) are released from alpha granules after the activation of platelets, which lead to leukocyte recruitment (Brandt et al., 2000; Frohlich, 2016). In addition, alpha granules also contain integral membrane protein P-selectin, which is translocated to the plasma membrane after the activation of platelets (Frenette et al., 2000). P-selectin glycoprotein ligand-1 (PSGL-1) expressed on leukocytes can interact with P-selectin and lead to the activation of neutrophils (Sreeramkumar et al., 2014). Furthermore, the released polyphosphates can activate Factor XII and lead to the initiation of the intrinsic coagulation pathway (Müller et al., 2009).

The activation of platelets can be determined according to ISO 10993-4 by measuring released contents from alpha granules, such as β-TG or PF4 using ELISA (Mayer et al., 2009; Teligui et al., 2016; Stoll et al., 2017) and detection of P-selectin (CD62P) or activated GPIIb/IIIa receptor with PAC-1 antibody using flow cytometry (Theoret et al., 2011; van Velzen et al., 2012).

## Activation of leukocytes

Besides coagulation, complement, and platelet activation, the activation of leukocytes and the occurrence of respiratory burst can be analyzed to evaluate biomaterial-induced inflammatory response. Thus, the generation of reactive oxygen species (ROS) and the release of PMN elastase are the mainly determined parameters for leukocytes activation. The activation of leukocytes leads to a respiratory burst (Dahlgren and Karlsson, 1999), which is the result of an enhanced oxygen metabolism, and results in generation of ROS (superoxide anion (${\text{O}}_{2}^{-}$), hydrogen peroxide (H_2_O_2_), hydroxyl radical (HO^∙^) and singlet oxygen (^1^O_2_). Thus, the ROS generation can be detected (Roesslein et al., 2013) using chemiluminogenic (Nygren et al., 2000) or fluorogenic substances (Ferrer et al., 2013). Furthermore, the release of elastase from PMN granulocytes, especially from neutrophils, can be quantified by ELISA (Zimmermann et al., 2007). Additionally, PMN elastase activity can also be measured by the proteolytic cleavage of a synthetic substrate and the release of a fluorophore, which can be easily quantified by fluorescence (Gramegna et al., 2017).

The activation of leukocytes leads to the increased expression of CD11b on the cell surface via translocation of the CD11b from intracellular granules to the plasma membrane. Thus, the detection of CD11b expression on the surface of leukocytes using fluorescence-activated cell sorting (FACS) can give additional information on activation of leukocytes (Gorbet et al., 1999). Furthermore, the production of neutrophil extracellular traps (NETs) is a recently described mechanism of neutrophils for host defense (Brinkmann et al., 2004; Delgado-Rizo et al., 2017). During NETosis, the nuclear material is released in form of a meshwork of chromatin by activated neutrophils to the extracellular space (Noubouossie et al., 2017). Several proteins adhere to the NETs, such as histones and components with antimicrobial activity, e.g., elastase and myeloperoxide (Delgado-Rizo et al., 2017). Besides the ability to trap bacteria, NETs ability to promote thrombosis was demonstrated in animal models (Brill et al., 2012).

## Analysis of biomaterial surfaces

The adsorption of proteins to the surface of biomaterials is the initial step directly after the blood contact. Subsequently, adhered proteins initiate the adhesion and activation of platelets on the surface. These platelets can further activate neighbored platelets and at the last step the thrombi are stabilized by the generation of a fibrin network.

The activation of platelets is a very fast process of ~180 ms (van Oeveren, 2013) and different morphological appearance of platelets can be detected on material surfaces depending on varying states of activation: (1) unactivated platelets: round, discoid shaped without pseudopodia, (2) partially activated platelets: dendritic with early pseudopodia, (3) moderately activated platelets: spread-dendritic with irreversible long-dendritic extensions, (4) fully activated platelets: fully spread (Park et al., 1990).

**Figure 3:**
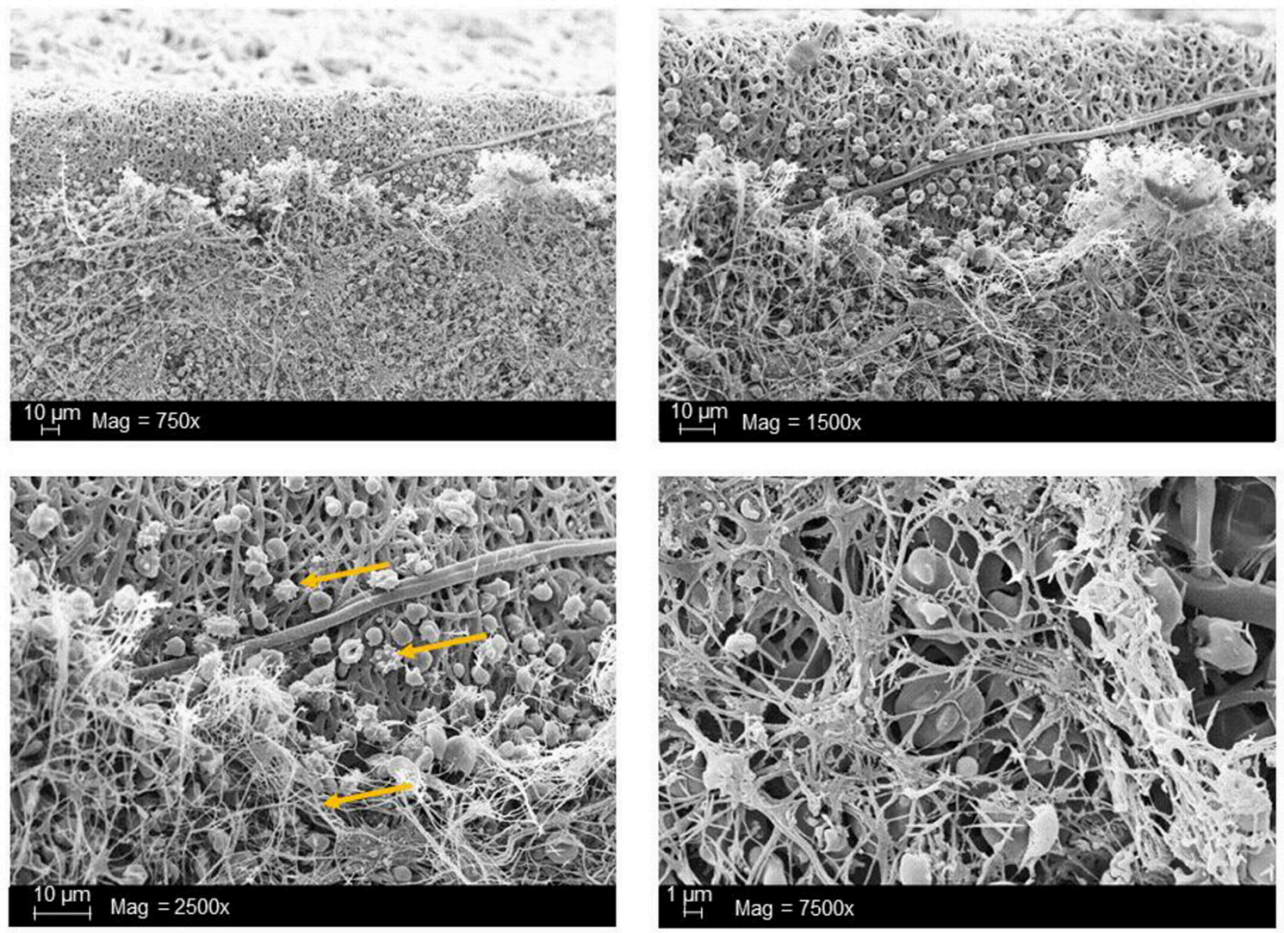
Scanning electron microscopic (SEM) analysis of synthetic vascular graft surface after the blood contact. Arrows indicate the adhered platelets as well as the resulting 3D-fibrin meshes due to activation of blood coagulation. The analyses were performed in our working group and the data has not been published before.

The adhesion and activation of platelets lead to a cytoskeletal rearrangement and therefore to a morphological change of platelets on the biomaterial surface with extensive formation of pseudopodia. Afterwards, the spreading of platelets and the release of vasoconstrictive substances, such as thromboxane and PDGF, as well as contents of stored granules occur. Finally, the aggregation of platelets and the generation of a fibrin network can be analyzed using scanning electron microscopy (SEM) (Zhang et al., 2017) (Figure 3). Thus, in several studies, the surface thrombogenicity of biomaterials was examined by characterization of cell morphology and spreading using SEM (Balasubramanian and Slack, 2001; Aguilar et al., 2002). Furthermore, using fluorescently labeled antibodies against specific receptors, the adhered cells and the cell density can be detected using fluorescence and confocal microscopy (Nguyen et al., 2016). In recent years, microgravimetric analyses using quartz crystal microbalance (QCM) were also applied to investigate the platelet adhesion and activation (Sinn et al., 2010; Fatisson et al., 2011; Kunze et al., 2014). Moreover, Hanson and colleagues used surface plasmon resonance (SPR) based flow chamber device to detect platelet-surface interactions and blood coagulation (Hansson et al., 2007). Furthermore, Zhao et al. (2011) used SEM and transmission electron microscopy (TEM) to investigate the effect of different sizes of nanoparticles on hemolysis and the mechanism behind the lysis of red blood cells. The group demonstrated that a small proportion of small type of mesoporous silica nanoparticles (MSNs) adsorbed to the surface of erythrocytes without any alteration of cell membrane or morphology. In contrast, the adsorption of large type MSNs to the erythrocytes induced a strong local membrane deformation and resulted in internalization of particles and hemolysis.

## Conclusion

The interaction of biomaterials with blood leads to cellular as well as humoral reactions, which can result in an unwanted inflammation and activation of coagulation and/or fibrinolysis. Thus, the development of biomaterials with an improved hemocompatibility increases the tolerability and minimizes unwanted side effects, such as thrombus formation. Therefore, during the development of new blood-contacting medical devices, not only the mechanical and chemical characteristics should play an important role, but also the hemocompatibility. Furthermore, to prove the safety and reliability of new products, hemocompatibility analyses should include appropriate references and follow the ISO 10993-4 standard.

## Author contributions

MA-A is the corresponding author of this work. She developed the outline with HW and CS edited the paper. MW and MA-A wrote the manuscript. HS, SG, and LH revised the manuscript.

### Conflict of interest statement

The authors declare that the research was conducted in the absence of any commercial or financial relationships that could be construed as a potential conflict of interest.
